# Carbapenemase-producing bacteria recovered from Nairobi River, Kenya surface water and from nearby anthropogenic and zoonotic sources

**DOI:** 10.1371/journal.pone.0310026

**Published:** 2024-11-14

**Authors:** Rael J. Too, Samuel M. Kariuki, George C. Gitao, Lilly C. Bebora, Dixie F. Mollenkopf, Thomas E. Wittum

**Affiliations:** 1 Centre for Microbiology (KEMRI-CMR), Kenya Medical Research Institute, Nairobi, Kenya; 2 The University of Nairobi, Department of Veterinary, Pathology, Microbiology and Parasitology (UoN-Kenya); 3 The Ohio State University, Department of Veterinary Preventive Medicine (OSU-VPM, OH, USA); Chulalongkorn University Faculty of Medicine and King Chulalongkorn Memorial Hospital, THAILAND

## Abstract

Carbapenem-resistant bacteria (CRB) present a significant global public health concern. Sub-Saharan Africa has borne a heavy burden of CRB with a reported prevalence of up to 60% in some patient populations. es in Africa focus on clinical CRB isolates, with limited data on their spread in the natural environment. Therefore, the purpose of this study was to report the recovery of CRB from Nairobi River surface waters and nearby anthropogenic and zoonotic sources in Nairobi County, Kenya. A total of 336 CRB were recovered from 336 (250 mL) samples, with 230 of the samples (68.5%) producing one or more CRB isolates. CRB were recovered most commonly from untreated sewage influent (100% of 36 samples; 79 total isolates), treated effluent (93% of 118 samples; 116 total isolates), Nairobi River surface waters upstream (100% of 36 samples; 57 total isolates), downstream (100% of 36 samples; 45 total isolates), and way downstream from the wastewater treatment plant (73% of 11 samples; 19 total isolates), slaughterhouse effluent discharges 1.5%, (5/336), animal contact areas 0.9%, (3/336), a manhole sewer from the affluent neighborhood of Karen at 2.7%, (9/336) respectively. The CRB included *Escherichia coli* (158, 47%), *Klebsiella pneumoniae* (74, 22%), and *Enterobacter spp* (43, 13%). *Aeromonas spp* (29, 9%) *Acinetobacter baumannii* (12, 3.6%), *Citrobacter freundii* (7, 2.1%), *Pseudomonas aeruginosa* (5, 1.5%) and other species (8, 2.4%). CRB genotypes included *bla*_NDM_ (246, 73.2%), *bla*_KPC_ (40, 12%), *bla*_VIM_ (51, 15.2%), *bla*_OXA-48-like_ (65, 19.3%), *bla*_IMP_ (15, 4.5%), and *bla*_GES_ (7, 2.1%). Sixty-nine of the CRB isolates (20.5%) harbored multiple carbapenemase-encoding genes. Our results indicate that clinically important CRB are commonly present in Nairobi River surface water and from nearby wastewater and livestock sources. These pose an important public health threat that requires urgent intervention strategies and additional investigation.

## Introduction

Carbapenem antimicrobials are generally reserved for use as a last option for the treatment of invasive multidrug-resistant bacterial infections in high-risk patients [1]. The emergence of carbapenem-resistant bacteria (CRB) represents an important public health threat that has been recognized by both the US Centers for Disease Control (CDC) [2] and the World Health Organization (WHO) [3]. CRB poses an important threat to high-risk patient populations, such as those housed in intensive care units, where case fatality rates as high as 50% have been reported [4]. In addition, healthcare environments including medical devices, equipment carts, wheelchairs and gurneys, and a wide variety of other surfaces may become contaminated with CRB and serve as fomites for transmission [4, 5]. Bacterial mechanisms of carbapenem resistance can include restriction of membrane permeability and cellular target alteration, but carbapenemase is the mechanism of primary epidemiologic importance. This is because carbapenemase production is frequently encoded on mobile genetic elements that are readily transferred within and between bacterial species. Individual carbapenemase genotypes that initially emerged regionally, including *bla*_NDM_ in Asia, *bla*_KPC_ in North America, and *bla*_VIM_ in Europe have subsequently disseminated globally. This global spread of CRB is not limited to healthcare environments, and healthy individuals in the community may become colonized following exposure to a variety of anthropogenic or zoonotic sources. These sources may include retail food products [6–9], agricultural [10–12] or companion animals [11–13], wastewater effluent [16, 17], or the natural environment including wildlife [18] and surface waters [9–22]. The epidemiology of CRB in the environment, including their main points of origination and impact, has not been fully elucidated. CRB has been reported in untreated wastewater influent, treated wastewater effluent, and surface waters near wastewater treatment plant discharge [17, 23–28], in many regions of the world, but data for Africa are not available. The prevalence of CRB among clinical isolates recovered from patients in Sub-Saharan Africa has been reported to be as high as 60% [29, 30]. A variety of globally disseminated CRB genotypes including *bla*_KPC_, *bla*_NDM_, *bla*_VIM_, *bla*_OXA-48-like_, and *bla*_IMP_ from *Pseudomonas aeruginosa*, *Klebsiella pneumoniae*, and *Acinetobacter baumannii* have been reported from various hospitals in East Africa, where CRB prevalence ranged from 22.4 to 34% [31–33]. However, the frequency that CRB escapes the healthcare environment and disseminates in surface waters in Africa is unknown.

We hypothesized that CRB present in human and animal waste in Nairobi County, Kenya routinely disseminate into the Nairobi River where they pose a risk to human and animal health. This study aims to report the frequency and recovery of CRB in the environment from Nairobi River surface water and from potential sources of dissemination including a wastewater treatment plant, a neighborhood sewer, and animal agriculture facilities in Nairobi County, Kenya.

## Materials and methods

### Description of study design and study area

This was a longitudinal study design where we systematically sampled multiple sites in the vicinity of Nairobi, Kenya including; the Ruai wastewater treatment plant (WWTP) located at: 1° 18’0” South, 36° 55’0” East [34], the Nairobi River near the WWTP effluent discharge sites, the Dagoretti slaughterhouse located at 00°4’7’S, 109° 11’41”E including its animal contact areas and wastewater treatment effluent, as well as influent and effluent from a manhole sewer in a nearby affluent neighborhood (Karen). The Ruai WWTP treats about 85,000 m^3^/day which represents approximately 80% of the wastewater generated from Nairobi County. The Dagoretti slaughterhouse serves as an economic hub for the people of Nairobi and its environs, where meat slaughtered from the abattoir is transported to Nairobi city and other nearby towns in Kenya. Different samples comprising raw untreated WWTP sewage influent, WWTP discharge final effluent, fecal samples from the animals grazing at the WWTP area, Nairobi River surface water at sites upstream, downstream, way downstream from the WWTP, slaughterhouse discharges and Swiffer samples from the wiped surfaces on animal contact areas including holding pens, hallways, and killing floor, cradles and hooks were aseptically collected once a week between the months of April 2021 and April 2022.

### Sampling strategy, sample collection, handling, and transportation

A systematic sampling technique was used to collect wastewater samples (250 mL) every Thursday of the week from the Ruai WWTP raw untreated influent and three fully treated effluent points (Effluent A, Effluent B, and Effluent C). Surface water samples (250 mL) were obtained from the Nairobi River at sites located upstream, downstream, and way downstream from WWTP effluent discharge sites at a distance of 100 meters apart all from the effluent discharge sites. Swiffer samples were collected from livestock stunning pens (lairage), hooks, killing areas, cradles, drainages, and other animal contact hallways at the slaughter facility. Samples of slaughterhouse effluent were collected from a drainage channel before discharge into the Kavuthi stream (a tributary of the Nairobi River). All samples were collected aseptically to avoid contamination and packaged in well-labeled sterile whirl-pak bags then placed into an ice cooler box and transported to the Kenya Medical Research Institute (KEMRI) Microbiology laboratory for processing.

### Sample processing and laboratory procedures

#### Filtration of water samples

To isolate CRB from water samples including influent and effluent, a membrane filter technique was used as previously described [34, 35]. The raw water samples collected in 250 mL polypropylene bottles were recorded and filtered through multiple cellulose membrane filters (Thermo Fischer Scientific™ Nalgene™ Filter Membranes) of different pore sizes to allow all the waters to pass through to avoid clogging utilizing a Millipore vacuum filtration system apparatus; starting with 41 μm filter paper, 20 μm, 10 μm, 1.2 μm 0.8 μm, and culminating with a 0.45 μm filter to capture bacteria [36]. All filters for a single sample were then placed into the same whirl-pak bag for culture.

#### Culture and isolation of carbapenemase-producing bacteria

Fifty mL of MacConkey (MAC) broth (BD) modified with 0.5 μg/mL meropenem and 70 μg/mL ZnSO4 (Oxoid Ltd) was added into whirl-pak bags containing filter papers used for filtration or/ and Swiffers used to wipe surfaces and fecal samples (5 g) which were then incubated aerobically for 18–24 hours at 37°C. After overnight growth, the suspension was inoculated onto MacConkey agar plates modified with 0.5 μg/mL meropenem and 70 μg/mL zinc sulfate and incubated for 18–24 hours at 37°C [37–39]. Based on morphological characteristics of Gram-negative bacteria and lactose fermentation, up to four pink colonies were picked for isolation from each sample plate and inoculated onto Mueller Hinton agar (MHA) modified with meropenem 0.5 μg/mL and 70 μg/mL ZnSO4 and incubated at 37°C for 24 hours [37]. Carbapenemase production was confirmed using the CarbaNP test [37] and matrix-assisted laser desorption/ionization (MALDI) time of flight (TOF) analyzer mass spectrometry (MS) was used to identify bacterial species where a MALDI target plate was spotted with a small amount of bacterial cell colony in the middle of both wells for that isolate ID with a toothpick. After putting all samples on each of their respective wells, 1μL of the bacterial test standard (BTS) on just the BTS well for quality control then dried at room temperature for a few minutes. Later the sample spot was overlayed with 1μL of formic acid onto each well (including the BTS well) and the plate was left to dry for several minutes. Matrix solution (10 mg/mL cyno-4-hydroxycinnamic acid in acetonitrile-water-trifluoroacetic acid (TFA) was vortexed for 3 minutes then, 1μL of the matrix liquid was put onto each well (including the BTS well) then left to dry prior to analysis.

#### Identification of CRB using the Carba-NP test directly from bacterial cultures

For Carba-NP, colonies from MHA agar were scraped off with a 1μL loop and suspended in two 1.5 mL Eppendorf tubes containing 100 μL of 20 mM Tris-HCl lysis buffer / Solulyse™ solution pH 7.8 already standardized then mixed [37, 40–42]. Standard control strains carrying *bla*_KPC_, and *bla*_NDM-1_ were included and incubated for 2 hours at 37°C based on the hydrolysis of the β-lactam ring of imipenem (Sigma) [37]. Thereafter, 15% glycerol stock was prepared from culture broths and Carba-NP positive isolates were preserved at -80°C for further analyses.

### Detection of carbapenemase-encoding genes

#### DNA extraction

 Bacterial DNA was extracted from CRB isolates by creating boiled lysates as previously described [43]. Isolates were emulsified in 200 μL nuclease-free water in a 1.5 mL Eppendorf tube placed in a hot plate at 100°C for 10 minutes and centrifuged at 16,000 rpm for 3 minutes to obtain the DNA template which was stored at -20°C until further analysis.

#### Control strains and primer sequences

The presence of specific carbapenemase-encoding genes including *bla*_KPC_, *bla*_NDM_, *bla*_IMP_, *bla*_OXA-48-like,_ *bla*_GES,_ and *bla*_VIM_ was determined using PCR for all phenotypic CRB isolates. Standard control strain (ATCC®BAA-1705™ *Klebsiella pneumoniae* ART 2008133 [D-05, 1338] *bla*_KPC_+*bla*_NDM_) was used as a positive control. The primer sequences were selected based on the specificity and sensitivity of the PCR product size distinguishable by agarose gel electrophoresis in multiplex PCR systems from different studies, described earlier [44–46] (Table 1).

**Table 1:** List of primer sequences used for screening carbapenemase-encoding genes.

Primer	Sequence (5’-3’)	Target gene	Amplicon size (bp)	References
**NDM1-F** **NDM1-R**	CAGCGCAGCTTGTCG TCGCGAAGCTGAGCA	*bla*_NDM_	784	[45]
**KPCyl-F** **KPCyl-R**	ATGTCACTGTATCGCCGTCT TTTTCAGAGCCTTACTGCCC	*bla*_KPC_	893	[43, 47, 48]
**VIM-F** **VIM-R**	AGTGGTGAGTATCCGACAG ATGAAAGTGCGTGGAGAC	*bla*_VIM_	261	[49]
**OXA-48F** **OXA-48R**	AAATCACAGGGCGTAGTTGTG GACCCACCAGCCAATCTTAG	*bla*_OXA-48- like_	555	[49]
**IMP-F** **IMP-R**	CATGGTTTGGTGGTTCTTGT ATAATTTGGCGGACTTTGGC	*bla*_IMP_	448	[49]
**GES-F** **GES-R**	CTTCATTCACGCACTATTAC TAACTTGACCGACAGAGG	*bla*_GES_	827	[50, 51]

#### Multiplex PCR for CRB genotype

Amplification reactions were performed [43, 47, 48] at a reaction volume of 25 μL. Three μL of template DNA was added to a master mix containing PuReTaq™ Ready-To-Go™ PCR beads (Cytiva, Global Life Sciences Solutions, Marlborough, MA, USA), 1.5 μL of forward and reverse primers, and 19 μL of nuclease-free water to a final volume of 25 μL.

The cycling conditions for all the PCR amplifications of carbapenem-resistant genes were as follows: 94°C for 5 minutes for initial denaturation and 30 cycles of 95°C for 30 seconds (denaturation), annealing at 52°C for 1 minute (for all primers except for *bla*_KPC_ which was at 58°C) and 72°C for 1 minute 30 seconds (extension), followed by 72°C for 10 minutes (final extension); performed in 0.2 mL Eppendorf PCR tubes in a thermal cycler (MJ Research PTC-200). Amplified PCR products were separated and visualized by electrophoresis using a 1% agarose tris-acetate-EDTA gel containing 0.5 μg EtBr staining. A 100–1,000 bp DNA ladder (Fermenters) was used to determine molecular weight.

#### Data management and statistical analysis

Using R- software version 4.2.1, data were analyzed using descriptive statistics using frequencies and percentages of CRB recovery computed as proportions of all samples collected and isolates recovered and presented using frequencies and percentages.

#### Ethical considerations

Study approval was sought from the Center Scientific Committee, Center for Microbiology Research (CSC-CMR), and Scientific Ethical Review Unit (SERU)-KEMRI protocol number: KEMRI/SERU/CMR/0126/4092. The National Commission for Science Technology and Innovation (NACOSTI) identification number 522511 and License No: NACOSTI/P/20/7287, and the Faculty Committee at the Department of Veterinary Pathology, Microbiology and Parasitology of the University of Nairobi. This was an environmental study and no consent was required. Other Approvals to access the field sites were sought from the Nairobi water and sewerage company (NWSC) and the County Government of Kiambu Livestock, Fisheries and Veterinary Services.

## Results

### Identification and characterization of CRB in the environment

A total of 336 samples were collected during the study period, of which 157 were from the WWTP, 83 were from Nairobi River surface water, 74 were from the slaughterhouse, and 22 were from the affluent neighborhoods of Karen manhole sewer (Table 2). From these 336 samples, 230 (68.5%) yielded 336 CRB isolates, with some samples yielding as many as four phenotypically distinct CRB (Table 2). Samples from the WWTP influent and effluent were most likely to contain CRB (94%) while Swiffer samples from the slaughterhouse animal contact surfaces had the lowest frequency of CRB recovery (2%) (Table 2).

**Table 2:** Sample-wise distribution of CRB isolates recovered from 336 environmental samples from Nairobi County, Kenya.

Study Area	Sampling site	Samples collected (n)	CRB Positive samples n (%)	CRB isolates (n)
Ruai (WWTP)	Raw sewage (RS)	36	36 (100%)	79
	Effluent A	36	29 (81%)	30
	Effluent B	36	35 (97%)	39
	Effluent C	36	36 (100%)	47
	Livestock fecal samples	13	1 (8%)	1
Nairobi River	Upstream	36	36 (100%)	57
	Downstream	36	36 (100%)	45
	Way Downstream	11	8 (73%)	19
Dagoretti Slaughterhouse (GS)	Swiffer samples (holding pen, killing floor, cradles, and hooks)	48	1 (2%)	1
	Effluent discharges	26	3 (12%)	3
Affluent neighbourhood of Karen	KRIN (influent)	11	6 (55%)	9
	KROUT (effluent)	11	3 (27%)	6
Total		**N = 336**	**230**	**336**

### Frequency and distribution of CRB in study sites

Of the 336 CRB isolates, 158 (47.0%) were identified as *Escherichia coli*, 74 (22%) as *Klebsiella pneumoniae*, 43 (12.8%) as *Enterobacter* spp, 29 (8.6%) as *Aeromonas* spp., 12 (3.6%) as *Acinetobacter baumannii*, 7 (2.1%) as *Citrobacter freundii*, 5 (1.5%) as *Pseudomonas aeruginosa* and 8 (2.4%) were other species including *Plesiomonas shigelloides*, *Citrobacter brakii*, *Kluyvera cryocrescens*, *Klebsiella vericola*, *Wautersiella falsenii*, *Acinetobacter bereziniae*, and *Camamonas kerstersii*.

### Frequency and distribution of carbapenemase-encoding genes

*bla*_NDM_ either alone or with other carbapenemase encoding genes was the most frequently identified CRB genotype (n = 246, 73.2%), and was recovered from all the sampling sites. *bla*_OXA-48-like_ was present in 65 isolates (19.3%), *bla*_VIM_ was present in 51 isolates (15.2%), *bla*_KPC_ was present in 40 isolates (11.9%) *bla*_IMP_ was present in 15 isolates (4.5%), and *bla*_GES_ was present in 7 isolates (2.1%) (Table 3).

**Table 3:** Frequency and distribution of carbapenemase-encoding genes of CRB recovered from environmental samples collected in Nairobi County, Kenya. Some isolates had multiple carbapenemase genes present.

Sampling site	Carbapenemase gene					
	*bla*_NDM_	*bla*_KPC_	*bla*_VIM_	*bla*_OXA-48-like_	*bla*_IMP_	*bla*_GES_
	n (% of isolates)	n (% of isolates)	n (% of isolates)	n (%of isolates)	n (% of isolates)	n (%of isolates)
**Ruai (WWTP)**	**142 (42.3)**	**22 (6.5)**	**37 (11)**	**46 (13.7)**	**12 (3.6)**	**5 (1.5)**
Raw Sewage	55 (16.4)	15 (4.5)	18 (5.4)	18 (5.4)	5 (1.5)	3 (0.9)
Effluent A	17 (5.1)	4 (1.2)	5 (1.5)	13 (3.9)	2 (0.6)	1 (0.3)
Effluent B	31 (9.3)	2 (0.6)	6 (1.8)	6 (1.8)	1 (0.3)	1 (0.3)
Effluent C	38 (11.3)	1 (0.3)	8 (2.4)	9 (2.7)	4 (1.2)	0
Fecal Samples	1 (0.3)	0	0	0	0	0
**Nairobi River**	**92 (27.4%)**	**17 (5.1)**	**11 (3.3)**	**18 (5.4)**	**3 (0.9)**	**1 (0.3)**
Upstream	44 (13.1)	5 (1.5)	6 (1.8)	9 (2.7)	1 (0.3)	1 (0.3)
Downstream	35 (10.4)	8 (2.4)	5 (1.5)	6 (1.8)	1 (0.3)	0
Way Downstream	13 (3.9)	4 (1.2)	0	3 (0.9)	1 (0.3)	0
**Dagoretti slaughterhouse**	**4 (1.2)**	**0**	**0**	**0**	**0**	**0**
Effluent	3 (0.9)	0	0	0	0	0
Swiffer Samples	1 (0.3)	0	0	0	0	1 (0.3)
**Affluent Sewer-Karen**	**8 (2.4)**	**1 (0.3)**	**3 (0.9)**	**1 (0.3)**	**0**	**1 (0.3)**
KRIN (influent)	6 (1.8)	0	1 (0.3)	0	0	0
KROUT (effluent)	2 (0.6)	1 (0.3)	2 (0.6)	1(0.3)	0	1 (0.3)
**Total n = 336 (%)**	**246 (73.2)**	**40 (11.9)**	**51 (15.2)**	**65 (19.3)**	**15 (4.5)**	**7 (2.1)**

*bla*_NDM_ alone or together with other carbapenemase genes was present in 85.4% of *E*. *coli*, 67.6% of *K*. *pneumoniae*, 41.9% of *E*. *bugandensis*, 31% of *A*. *caviae*, and 36.4% of *C*. *freundii* and all other species. The distribution of CRB genotypes among the various bacterial species is summarized in Table 4.

**Table 4:** Frequency and distribution of carbapenemase encoding genes among species of CRB recovered from environmental samples collected in Nairobi County, Kenya.

Carbapenemase encoding genes						
Bacterial species	*bla*_NDM_	*bla*_KPC_	*bla*_VIM_	*bla*_OXA-48 like_	*bla*_IMP_	*bla*_GES_
***E*. *coli***	**135 (40.2)**	7 (2.1)	**11 (3.3)**	**8 (2.4)**	**6 (1.8)**	**1 (0.3)**
***K*. *pneumoniae***	**50 (14.9)**	6 (1.8)	**10 (3.0)**	**17 (5.1)**	3 (0.9)	
*A*. *baumannii*	10 (3.0)	1 (0.3)	1 (0.3)	1 (0.3)		
*P*. *aeruginosa*	1 (0.3)		2 (0.6)	3 (0.9)		
*E*. *asburiae*	1 (0.3)	2 (0.6)	1 (0.3)			
***E*. *bugandensis***	**18 (5.4)**	**8 (2.4)**	**13 (3.9)**	**16 (4.8)**	**3 (0.9)**	**3 (0.9)**
*E*. *cloacae*	4 (1.2)		1 (0.3)	5 (1.5)	1 (0.3)	1 (0.3)
*E*. *kobei*	3 (0.9)	2 (0.6)	3 (0.9)	5 (1.5)		1 (0.3)
***A*. *caviae***	**9 (2.7)**	**4 (1.2)**	**5 (1.5)**	**4 (1.2)**	**1 (0.3)**	**1 (0.3)**
*A*. *hydrophila*	2 (0.6)	1 (0.3)				
*A*. *veronii*	4 (1.2)	6 (1.8)	1 (0.3)	1 (0.3)	1 (0.3)	
*A*. *balyai*	1 (0.3)					
*A*. *bereziniae*		1 (0.3)		1 (0.3)		
*C*. *freundii*	4 (1.2)			3 (0.9)		
*C*. *kerstersii*	1 (0.3)					
*K*. *cryocresence*	1 (0.3)	1 (0.3)	1 (0.3)			
*K*. *vericola*	1 (0.3)		1 (0.3)			
*P*. *shigelloides*		1 (0.3)	1 (0.3)	1 (0.3)		
*W*. *falsenii*	1 (0.3)					
**Number of occurrences (n = 336) (%)**	**246 (73.2)**	**40 (11.9)**	**51 (15.2)**	**65 (19.3)**	**15 (4.5)**	**7 (2.1)**

* Some isolates had multiple carbapenemase encoding genes present making the total number of carbapenemase genes greater than the total number of CRE isolates.

Sixty-nine of the CRB isolates (20.5%) harbored multiple carbapenemase-encoding genes (Table 5). The majority (41, 59.4%) of these isolates carried 2 genes, while (22, 31.9%) carried 3, and (6, 8.7%) carried 4 carbapenemase-encoding genes (Table 5). The most frequently observed combination CRB genotype was *bla*_NDM_ + *bla*_VIM_ carried by eleven (15.9%) isolates (Table 5).

**Table 5:** Frequency of multiple carbapenemase-encoding genes present in individual CRB isolates recovered from environmental samples in Nairobi County, Kenya.

Multiple carbapenemase genes	Total	Carbapenem-resistant bacteria
*bla*_NDM +_ *bla*_VIM_	11	*E*. *coli*, Enterobacter spp.
*bla*_KPC +_ *bla*_VIM +_ *bla*_OXA-48_	7	*E*. *coli*, *K*. *pneumoniae*, *A*. *caviae*, *E*. *kobei*, *P*. *shigelloides*
*bla*_NDM +_ *bla*_OXA-48_	7	*E*. *bugandensis*, *E*. *cloacae*, *K*. *pneumoniae*
*bla*_KPC +_ *bla*_OXA-48_	6	*E*. *coli*, *K*. *pneumoniae*, *E*. *bugandensis*, *A*. *bereziniae*,
*bla*_VIM +_ *bla*_OXA-48_	5	*K*. *pneumoniae*, *A*. *baumannii*, *Enterobacter spp*., *P*. *aeruginosa*
*bla*_NDM +_ *bla*_VIM +_ *bla*_OXA-48_	4	*E*. *bugandensis*
*bla*_NDM +_ *bla*_VIM +_ *bla*_KPC_	3	*E*. *coli*, *E*. *bugandensis*, *K*. *cryocresence*
*bla*_OXA-48 +_ *bla*_IMP_	2	*K*. *pnemoniae*, *Enterobacter spp*.
*bla*_OXA-48 +_ *bla*_IMP +_ *bla*_IMP-27_	2	*E*. *coli*, *K*. *pneumoniae*, *Enterobacter spp*.
*bla*_KPC +_ *bla*_VIM_	2	*Aeromonas spp*. *Enterobacter* spp.
*bla*_NDM +_ *bla*_IMP_	2	*E*. *bugandensis*, *A*. *veronii*
*bla*_NDM +_ *bla*_KPC +_ *bla*_OXA-48_	2	*Enterobacter spp*.
*bla*_NDM +_ *bla*_KPC +_ *bla*_VIM +_ *bla*_OXA-48_	2	*Enterobacter spp*.
*bla*_IMP_ *+ bla*_IMP-27_	1	*E*. *coli*
*bla*_IMP +_ *bla*_GES_	1	*E*. *cloacae*
*bla*_VIM_ *+ bla*_GES_	1	*E*. *cloacae*
*bla*_VIM +_ *bla*_IMP_	1	*E*. *coli*
*bla*_VIM +_ *bla*_OXA-48 +_ *bla*_GES_	1	*E*. *bugandesis*
*bla*_KPC +_ *bla*_VIM +_ *bla*_IMP_	1	*A*. *baumannii*
*bla*_KPC +_ *bla*_VIM +_ *bla*_OXA-48 +_ *bla*_GES_	1	*A*. *caviae*
*bla*_NDM +_ *bla*_IMP-27_	1	*E*. *coli*
*bla*_NDM +_ *bla*_OXA-48 +_ *bla*_IMP-27_	1	*A*. *veronii*
*bla*_NDM +_ *bla*_OXA-48 +_ *bla*_IMP +_ *bla*_GES_	1	*E*. *coli*
*bla*_NDM +_ *bla*_VIM +_ *bla*_IMP_	1	*E*. *bugandensis*
*bla*_NDM +_ *bla*_VIM +_ *bla*_IMP +_ *bla*_IMP-27_	1	*E*. *bugandensis*
*bla*_NDM +_ *bla*_VIM +_ *bla*_OXA-48 +_ *bla*_IMP_	1	*A*. *veronii*
*bla*_NDM +_ *bla*_KPC_	1	*Enterobacter spp*.
**Total**	**69**	

## Discussion

We observed a high frequency of CRB from environmental samples from surface waters of the Nairobi River, and from nearby sources including Ruai WWTP influents and effluents, and Dagoretti slaughterhouse discharges that yielded multiple bacterial species expressing both phenotypic and genotypic carbapenem resistance, our results are consistent with other studies [39, 52, 53] where environmental samples showed 88% and 50% phenotypically confirmed carbapenemase-producing strains. We identified CRB producing a variety of different carbapenemase enzymes detected from wastewater and swiffer samples from different environmental niches.

The organisms that we recovered included *Enterobacter* spp., *K*. *pneumoniae*, *A*. *baumannii*, and *P*. *aeruginosa*, which the WHO had designated as being of critical importance. However, we also recovered *E*. *coli* and *Aeromonas* spp., which have been reported in hospitals, WWTPs, farms, and other environments [24, 53, 54]. While the majority of scientific literature focuses on clinical CRB isolates, *E*. *coli* is the most frequently recovered CRB from environmental samples as a result of being a conserved bacteria [55], and its ability to exchange resistance genes via horizontal gene transfer [56].

The *bla*_NDM_ genotype was the most frequently detected among the CRB. The majority of the *E*. *coli* and some *K*. *pneumoniae* isolates carried *bla*_NDM_ alone or co-harbored with other carbapenemase genes. This result is not unexpected because Africa accounts for approximately 10.8% of all *bla*_NDM-1_ cases reported worldwide [57]. The most common carbapenemase enzymes reported in clinical isolates across all African countries are NDM-type and OXA-type [30]. *bla*_NDM_ was first reported in Kenya in 2007, originally from *K*. *pneumoniae* and later from an *A*. *baumannii* hospital outbreak [31, 58]. However, there is a lack of data on CRB from environmental sources in Kenya including surface water. Previous reports of clinical CRB in Kenya identified *K*. *pneumoniae* and *E*. *coli* harboring *bla*_NDM_ and *bla*_OXA_ alleles [59, 60]. Our results suggest that in Kenya, CRB has spread from hospital settings into the environment as has been reported in other countries [26].

Among the CRB, *K*. *pneumoniae* and *E*. *coli* have been highlighted by the WHO in their priority list of pathogens [61, 62]. Numerous studies have demonstrated the international spread of CRB genotypes, most commonly *bla*_KPC_ and *bla*_NDM_. In Egypt [63], clinical CRB isolates harboring *bla*_OXA-48-like_ have been reported. In addition, others [24, 54, 64] have reported CRB harboring *bla*_OXA-48-like_, *bla*_NDM_, and *bla*_KPC_ in environmental niches, including WWTP in Eastern Cape Province, South Africa. A similar observation of a low prevalence of *bla*_NDM-1_, *bla*_NDM-7,_ and *bla*_OXA-181_-producing *E*. *coli* clinical isolates in Iran has been reported [65]. Our results emphasize the potential importance of environmental CRB in Sub-Saharan Africa and suggest the need for greater research in this area.

It is noteworthy that 2475 tons of trash are created in Nairobi daily, and the majority of it is dumped directly into the Nairobi River from both industrial activity and informal settlements located around Nairobi County [66]. Consequently, the Nairobi River is heavily contaminated with untreated industrial and human garbage. This waste may be a source of chronic selective pressure exerted by antibiotics and other chemical pollutants including heavy metals such as lead and mercury [67]. Prior research [68–71] has primarily focused on antimicrobial residues in wastewater and water surfaces. Our results suggest that the dissemination of antimicrobial-resistant bacteria including CRB in the natural environment of Kenya, including the Nairobi River, is an important public health threat as well. Recent studies have reported clinical CRB similar to the CRB in our study [1, 33, 72, 73]. This suggests that hospitals and other healthcare settings may be the original source of the CRB recovered from the natural environment. Furthermore, the presence of CRB in the manhole sewer of the affluent neighborhoods of Karen and from the slaughterhouse suggests community transmission of CRB. This is an important public health concern for Kenya and all of Africa.

In summary, the presence of a wide variety of CRB from the Nairobi River in Kenya, and from nearby anthropogenic and zoonotic sources of contamination provides evidence that CRB originating in healthcare environments can disseminate in the community and into natural environments. Our research demonstrates the ability of environmental and wastewater AMR surveillance to identify epidemiologically important strains and clinically relevant antimicrobial resistance genes in Africa. Based on this information, control measures can be taken to reduce the risk to public health including proper waste treatment, both onsite at healthcare facilities and the final effluents from WWTPs before they are discharged into the receiving environment.
